# Testing on bacterial vaginosis in a subfertile population and time to pregnancy: a prospective cohort study

**DOI:** 10.1007/s00404-024-07542-x

**Published:** 2024-05-16

**Authors:** Marjolein van den Tweel, Ellen van den Munckhof, Moniek van der Zanden, Saskia Le Cessie, Jan van Lith, Kim Boers

**Affiliations:** 1 Department of Obstetrics and Gynecology, Leiden University Medical Center, 2300RC Leiden, The Netherlands; 2 Department of Obstetrics and Gynecology, Haaglanden Medical Center, 2597AX The Hague, The Netherlands; 3 DDL Diagnostic Laboratory, 2288ER Rijswijk, The Netherlands; 4 Department of Biomedical Data Sciences, Leiden University Medical Center, 2300RC Leiden, The Netherlands; 5 Department of Clinical Epidemiology, Leiden University Medical Center, 2300RC Leiden, the Netherlands

**Keywords:** Bacterial vaginosis, qPCR, Subfertility, Time to pregnancy, Microbiome

## Abstract

**Purpose:**

This study aimed to investigate the influence of bacterial vaginosis on time to pregnancy in subfertile couples.

**Methods:**

Couples attending a teaching hospital in the Netherlands having an initial fertility assessment (IFA) between July 2019 and June 2022 were included in this prospective study, with follow-up of pregnancies until June 2023. Vaginal samples at IFA were analyzed on pH, qPCR BV, and 16S rRNA gene microbiome analysis of V1-V2 region. Main outcome measures were time from initial fertility assessment to ongoing pregnancy at 12 weeks and live birth, analyzed by Kaplan–Meier and Cox regression with adjustment for potential confounders.

**Results:**

At IFA, 27% of 163 included participants tested positive for BV. BV status had no influence on time to ongoing pregnancy (HR 0.98, 0.60–1.61, aHR 0.97, 0.58–1.62). In persons with unexplained subfertility, positive BV status had a tendency of longer time to pregnancy. When persons had an indication for fertility treatment, positive BV status (HR 0.21, 0.05–0.88, aHR 0.19, 0.04–0.85) and microbiome community state type III and type IV had significant longer time to pregnancy.

**Conclusion:**

This study indicates that BV may have a potential negative impact on time to live birth pregnancy in subfertile persons with an indication for fertility treatment. This study did not find an association between BV and time to live birth pregnancy in a general group of subfertile couples or in unexplained subfertility. More research should be done in persons with unexplained subfertility and if treatment improves time to pregnancy.

**Supplementary Information:**

The online version contains supplementary material available at 10.1007/s00404-024-07542-x.

## What does this study add to the clinical work

**Table:** 

This study indicates that bacterial vaginosis may have a potential negative impact on time to live birth pregnancy only in subfertile persons with a low chance of spontaneous pregnancy.	

## Introduction

Up to one in eight couples experience subfertility, and among them, 25% will be diagnosed with unexplained subfertility [1]. Subfertility could form a psychologic burden on couples, but also poses an economic burden on society due to substantial costs [2, 3].

In the Netherlands, couples are typically referred for an initial fertility assessment (IFA) with a gynecologist after experiencing one year of subfertility. During the IFA, the Hunault prediction model is used to determine whether fertility treatment is necessary or expectant management is a viable option [4]. Expectant management is often chosen for unexplained subfertility.

A possible link between unexplained subfertility and bacterial vaginosis (BV) has been mentioned in previous studies [5]. Bacterial vaginosis (BV) refers to a dysbiosis of the vaginal microbiome. While BV can cause discharge problems with a fishy odor, approximately half of BV-positive persons are asymptomatic. Several studies have shown an incidence rate of 10–32% of BV, with a higher occurrence among subfertile women and certain ethnicities [6, 7]. The diagnosis of BV can be made using the Nugent score, but qPCR has been shown to be more sensitive in detecting BV [8]. However, qPCR and microbiome testing have so far mainly be used in IVF populations to investigate the impact of BV on pregnancy rates. In the IVF population, BV was associated with early pregnancy loss and lower clinical pregnancy rates [9]. Only two studies have investigated BV in a preconception population cohort and found a probable effect on fecundity using the Nugent score or a 16S rRNA gene microbiome analysis to diagnose BV [10, 11].

Metronidazole and clindamycin are commonly used therapies for BV, but the recurrence rate within a year remains high [12]. Certain lifestyle factors have been associated with BV [13], which might influence the treatment of BV as well as pregnancy outcomes. It is also found that BV changes over time, and timing of fertility treatments is crucial [14]. However, the exact causal relation between BV and subfertility is still unknown, and it remains uncertain which approach improves outcomes. Having a more precise understanding of the direct correlation between BV and pregnancy outcomes may offer insights for potential future treatments.

This study investigates the effect of BV in a subfertile population diagnosed by qPCR at the time of initial fertility assessment on time to live birth pregnancy. It is hypothesized that persons who test positive for BV may experience a longer time interval to pregnancy, particularly in participants with unexplained subfertility or those with a direct indication for fertility treatments such as IUI or IVF/ICSI.

## Materials and methods

### Patient recruitment, sample, and data collection

This is a prospective single-center cohort study in the Haaglanden Medical Center (HMC, a teaching hospital) in The Hague, the Netherlands. Persons above 18 years old were included after informed consent at the initial fertility assessment (IFA) between July 2019 and June 2022. Persons were excluded when they had a history of three or more miscarriages, could not understand Dutch or English, or used prophylactic antibiotic treatment. This study was designed as part of a prospective cohort study about BV and pregnancy results during IUI and IVF/ICSI treatment (van den Tweel 2023 [15]); therefore, no separate power analysis was conducted.

The vaginal swab (e-swab, Copan Italia SpA, Breschia, Italy) and pH measurement (pH-Fix 4.0–7.0, ref 92137, Macherey–Nagel, Düren, Germany) were taken from the posterior fornix after inserting a speculum. Assessment of vaginal swabs for BV was done by external laboratories NMDL and DDL, Rijswijk, The Netherlands. Study participants and their doctors were blinded for the outcome of the swab. If study participants had symptoms of BV at any point, they underwent additional testing according to the standard protocol and treated (with clindamycin or metronidazole) if they tested positive for BV.

Follow-up ended at the point of not wishing to conceive, live birth, end of relationship, age 43 years old or end of study (October 2022). Follow-up of established pregnancies in this period was continued until it was known whether the pregnancy resulted in a live birth. Information about patient characteristics, fertility treatment, and pregnancy outcomes were collected from the electronic patient dossiers. To minimize loss to follow-up, a survey was sent to study participants if they did not return to the fertility clinic or gave birth elsewhere. This information was managed using Castor EDC, a cloud-based clinical data management service.

### BV qPCR and microbiome analysis

The vaginal samples were frozen within 24 h after collection and transported to the external laboratory (NMDL and DDL, Rijswijk, the Netherlands) for molecular analysis. DNA was extracted from 200 µL sample and eluted in a final volume of 100 µL with the MagNA Pure 96 instrument using the MagNA Pure 96 DNA and Pathogen Universal small Volume Kit and the Pathogen Universal protocol (Roche Diagnostics, Basel, Switzerland). The extracted DNA of all obtained vaginal swabs was tested with a CE-IVD marked multiplex quantitative PCR assay, the AmpliSens® Florocenosis/Bacterial vaginosis-FRT PCR kit (InterLabService, Moscow, Russia) according to the manufacturer’s instructions. Based on the presence of *Lactobacillus* species, *Gardnerella vaginalis*, *Atopobium vaginae* (recently reclassified as *Fannyhessea vaginae*) [16] and total amount of bacteria, swabs were categorized as BV positive (amount of *G. vaginalis* and/or *A. vaginae* is almost equal or exceeds the amount of *Lactobacillus* spp.), BV negative (*G. vaginalis* and/or *A. vaginae* are absent or its amount is substantially less than the *Lactobacillus* spp. amount), unspecified dysbiosis (amount of *Lactobacillus* spp. is reduced relative to the total amount of bacteria, whereas *G. vaginalis* and/or *A. vaginae* are absent or its amount is substantially less than total amount of bacteria) or suspected dysbiosis (amount of *G. vaginalis* and/or *A. vaginae* is similar to the amount of *Lactobacillus* spp. but does not exceed the limit value) using the software tool provided by the kit manufacturer. Unspecified dysbiosis and suspected dysbiosis were classified as BV qPCR positive.

In a subgroup of participants, the microbiota composition was determined. A fragment of ~ 421 bp of the V1-V2 region of the 16S rRNA gene was amplified using the primers described by Ravel, et al. (2011) and Walker, et al. (2015) with Illumina overhang adaptor sequences added [17, 18]. Results were classified in one of five vaginal microbiome community state types (CST), as described by Ravel et al. CST I is dominated by *L. crispatus*, and, respectively, CST II by *L. gasseri*, CST III by *L. iners*, CST IV by non-lactobacilli, CST V by *L. jensenii*. More detailed information on the microbiota analysis is described in attachment 1.

### Outcomes

Primary endpoint of the study was time until live birth pregnancy (time calculated from date of initial fertility assessment swab until positive pregnancy test leading to live birth). Secondary endpoints were time until ongoing pregnancy rate at 12 weeks’ gestation, miscarriage, and preterm birth rates.

### Statistical analysis

IBM SPSS statistics version 27 was used for all analysis. Continuous variables were compared between participants with and without BV using an unpaired *t *test or a Mann–Whitney U test in case of skewed distributions. Categorical variables are compared using the Chi^2^ or Fischer’s exact test. Kaplan–Meier curves with the log rank test were used to compare the time to pregnancy between the two groups. Time to pregnancy was measured between date of IFA swab until date of positive pregnancy test. We performed Cox proportional hazard analysis to analyze time to pregnancy and to adjust for BMI and age which were considered the most important confounders. Because of low number of pregnancies, it was not possible to adjust for more than two confounders. Subgroup analysis was performed for unexplained fertility, Caucasian descent, and direct indication for IUI or IVF/ICSI treatment, because literature suggests a different impact of BV in these groups.

## Results

### Study population

**Fig. 1:**
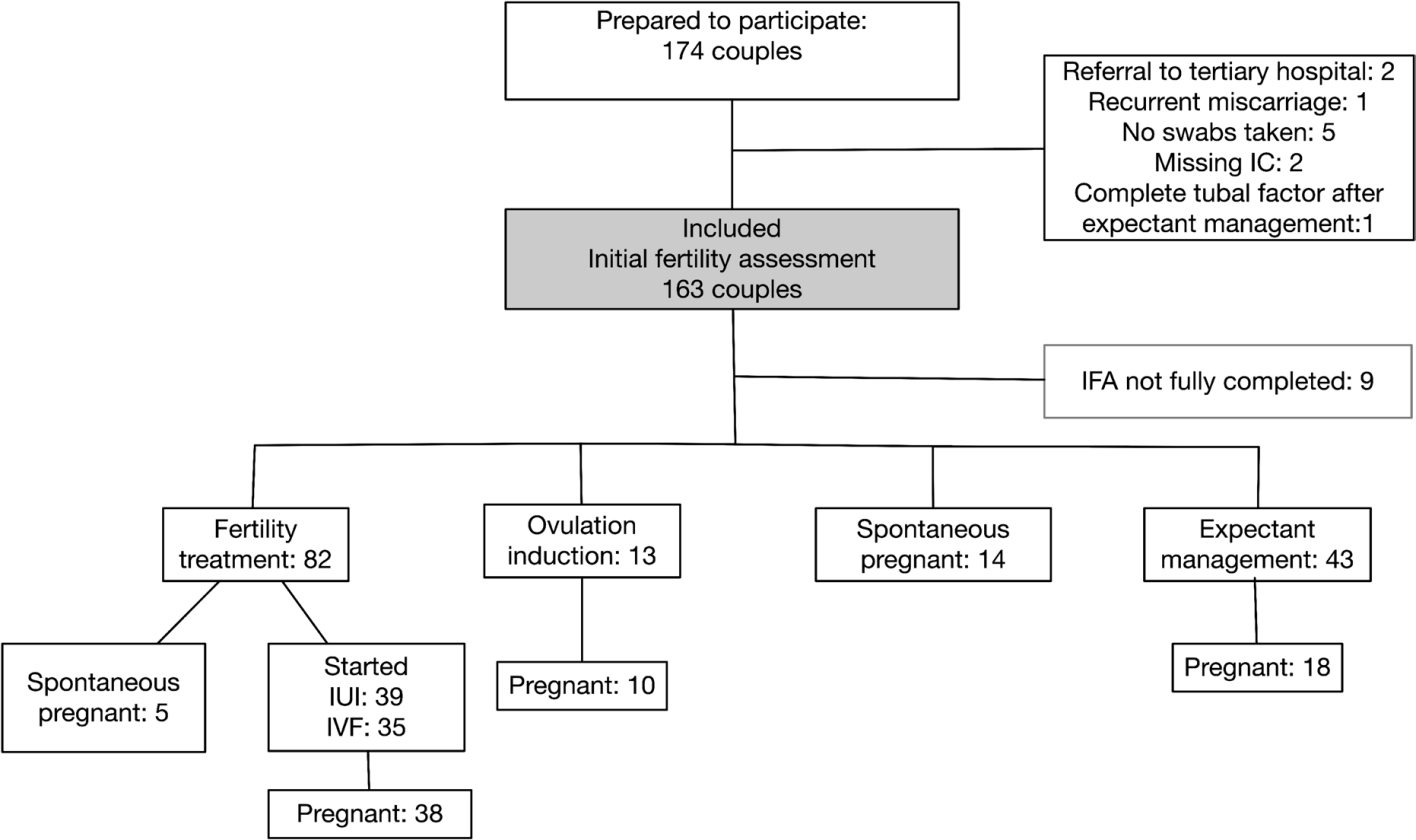
Flow diagram of included couples and all first follow-up treatments

A total of 163 persons were included at the initial fertility assessment (Fig. 1). Eighty-two of included participants needed direct IUI or IVF/ICSI treatment. One person was excluded because after follow-up of expectant management, a complete tubal factor was encountered. Fourteen participants conceived during their initial fertility assessment work up. Eighty-three of the included couples eventually had a live birth.

Of the total of 163 participants, 27% tested positive on BV qPCR at initial fertility assessment (Table 1). For one participant, no qPCR result was available at IFA, but vaginal swabs collected during two follow-up treatments were tested BV qPCR negative. Therefore, this participant was regarded as BV qPCR negative. Maximum age at IFA was 42 years. BMI and pH were significantly higher in the BV qPCR positive group. BMI was not available for seven participants. Twelve participants reported discharge problems. Three of them were treated with antibiotics for, respectively, a yeast infection after hysterosalpingogram, BV or for urinary tract infections. The duration of subfertility of BV qPCR positive participants was slightly longer before visiting the outpatient clinic (difference of 6 months, *p* = 0.09). Male factor was more often the reason of subfertility in BV qPCR negative participants (25% vs. 12%) and participants with endometriosis never tested BV qPCR positive, while BV qPCR positive participants had more often a hormonal factor or tubal factor as reason for subfertility. The follow-up survey was sent to 50 participants, of which 40 replied (which included all pregnant participants).

**Table 1:** Baseline characteristics

Baseline IFA	BV pos	BV neg	*p* value
Participants	44	119	
Age (at IFA) (mean, SD)	33 (4.0)	34 (4.4)	0.27
pH (median, IQR)	5.5 (5.0–5.8)	4.4 (4.0–4.7)	< 0.001^a^
Discharge complaints (at IFA) *n* (%)	4 (9%)	8 (7%)	0.52
Antibiotic/antifungal treatment	1	2	1.00
BMI (at IFA) (mean, SD)	26 (4.8)	24 (4.0)	0.03^a^
Smoking (at IFA) *n* (%)	8 (18%)	11 (9%)	0.11
Alcohol (≥ 1 glas per week) *n* (%)	14 (32%)	44 (37%)	0.52
Drug use (on regular basis) *n* (%)	4 (9%)	5 (4%)	0.26
Medication use *n* (%)	5 (11,5%)	28 (24%)	0.09
Chlamydia antibodies positive *n* (%)	6 (14%)	14 (12%)	0.74
Ethnicity, *n* (%)b			0.11
Caucasian	22 (50%)	78 (65%)	
African	1 (2%)	2 (1,5%)	
Antillean	7 (16%)	3 (2,5%)	
Asian	2 (5%)	7 (6%)	
Moroccan	2 (5%)	7 (6%)	
Hindu	5 (11%)	8 (7%)	
Turkish	0	3 (2,5%)	
Other	4 (9%)	9 (8%)	
Missing	1 (2%)	2 (1,5%)	
Socioeconomic status, *n* (%)c			0.14
Low	4 (9%)	4 (3%)	
Middle	13 (30%)	31 (26%)	
High	25 (56%)	83 (70%)	
Missing	2 (5%)	1 (1%)	
Regular cycle *n* (%)	35 (80%)	101 (85%)	0.42
HPV positive last year, *n* (%)	4 (9%)	13 (11%)	1.00
Gravidity, *n* (%)			0.72
0	26 (59%)	69 (58%)	
1	10 (23%)	35 (29%)	
2	7 (16%)	13 (11%)	
3	1 (2%)	1 (1%)	
5	0	1 (1%)	
History of preterm birth	0	4	0.57
History of C-section	1	9	0.27
Subfertility duration in years (median, IQR)	2 (1–2)	1.5 (1–2)	0.09
Cause of subfertility, *n* (%)d			0.07
Male factor	5 (11,5%)	29 (24,5%)	
Tubal factor	5 (11,5%)	7 (6%)	
Hormonal	11 (25%)	17 (14%)	
Endometriosis	0 (0%)	10 (8,5%)	
Unexplained	19 (43%)	48 (40%)	
Other	1 (2%)	5 (4%)	
Missing	3 (7%)	3 (3%)	
First treatment, *n* (%)			0.29
Expectant management	9 (20,5%)	34 (29%)	
Spontaneous pregnancy	9 (20,5%)	15 (13%)	
IUI	11 (25%)	28 (23%)	
IVF	6 (14%)	29 (24%)	
Ovulation induction	5 (11%)	8 (7%)	
IFA not finished	4 (9%)	5 (4%)	

^a^*p* value < 0.05 considered significant

^b^Other mostly Hispanic participants

^c^As defined by education status

^d^Hormonal: premature ovarian insufficiency or anovulation, other: uterine myomas, uterus anomaly, sexual disfunction

### Pregnancy results

**Fig. 2:**
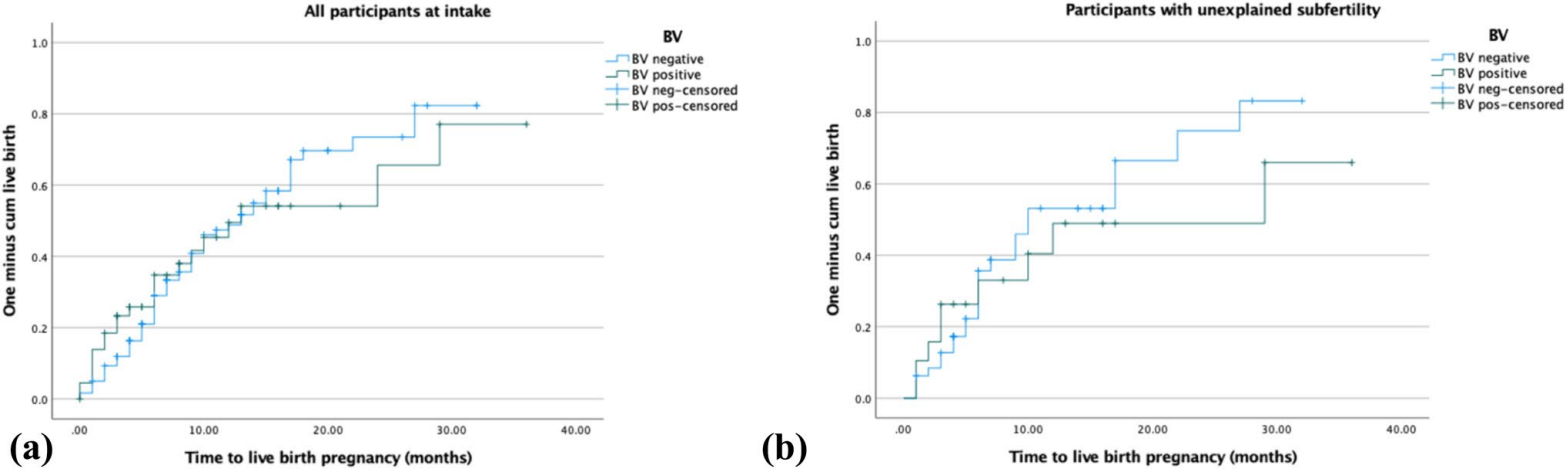
Kaplan–Meier curves for time to live birth pregnancy based on qPCR status at IFA for **a** all participants and **b** participants with unexplained subfertility

Out of 163 couples, 85 had an ongoing pregnancy (Table 2). Median follow-up time without an occurring pregnancy was 8 months (IQR 5–16). No significant differences were found for BV qPCR status on time to ongoing pregnancy rates (aHR 0.94, 0.57–1.57) or time to live birth pregnancy rates (aHR 0.97, 0.58–1.62) (Table 2 and Fig. 2a). Two participants had a termination of pregnancy because of Noonans syndrome or because of a serious neural tube defect. Twelve participants (two (5%) BV qPCR pos and ten (8%) BV qPCR neg) delivered prematurely, of which eleven between 31- and 37-weeks’ gestation, and one participant delivered at 24 weeks pregnant, after which the newborn died 7 days postpartum due to its prematurity. The two premature deliveries in the BV qPCR positive group occurred at 36 weeks.

**Table 2:** Pregnancy results by BV qPCR status

Outcomes	BV pos	BV neg	HR (CI 95%)	*p* value	aHR (CI 95%)^b^
Participants	44	119			
Ongoing pregnancy, *n* (%)	21 (48%)	64 (54%)	0.95 (0.58–1.56)	0.85	0.94 (0.57–1.57)
Live birth, *n* (%)	21 (48%)	62 (52%)	0.98 (0.60–1.61)	0.94	0.97 (0.58–1.62)
Premature birth, *n* (%)	2 (5%)	10 (8%)			
Miscarriage in follow-up period^a^	7 (16%)	22 (18%)		0.70	
Persons with unexplained subfertility	19	48			
Ongoing pregnancy, *n* (%)	9 (47%)	24 (50%)	0.78 (0.35–1.70)	0.52	0.63 (0.28–1.45)
Live birth, *n* (%)	9 (47%)	24 (50%)	0.78 (0.35–1.70)	0.52	0.63 (0.28–1.45)
Miscarriage in follow-up period^a^	5 (26%)	8 (17%)		0.37	
Persons starting IUI/IVF	21	61			
Ongoing pregnancy, *n* (%)	8 (38%)	35 (57%)	0.52 (0.23–1.17)	0.11	0.49 (0.20–1.15)
Live birth, *n* (%)	8 (38%)	33 (54%)	0.54 (0.24–1.23)	0.14	0.50 (0.21–1.19)
Miscarriage in follow-up period^a^	5 (24%)	14 (23%)		0.94	
Ongoing pregnancy/live birth (from 6 months onwards)			0.21 (0.05–0.88)	0.03^a^	0.19 (0.04–0.85)

^a^Persons experiencing one or more miscarriages

^b^Adjusted for BMI and age

In the subgroup of 67 participants with unexplained fertility, a tendency to a longer time to live birth pregnancy was observed in the BV qPCR positive group, but differences were not statistically significant (aHR 0.63, 0.28–1.45) (Fig. 2b). Data about only Caucasian or non-Caucasian population and time to pregnancy based on BV status did not show any differences (see Supplement Fig. S4a,b and Supplemental Table S3).

### Pregnancy results in participants with a direct IUI or IVF/ICSI indication

**Fig. 3:**
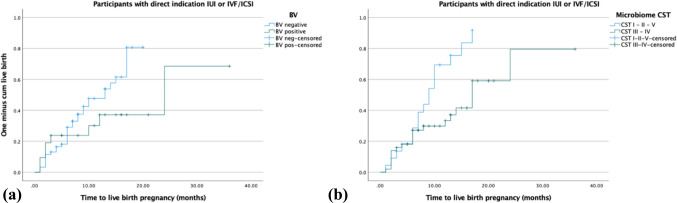
Kaplan–Meier curves for time to live birth pregnancy when IUI or IVF/ICSI treatment is indicated by (a) BV qPCR and (b) microbiome analysis

Eighty-two participants had an indication of treatment by IUI or IVF/ICSI based on the IFA. The Kaplan–Meier curve showed longer duration until live birth pregnancy in the BV qPCR positive group (Fig. 3a) in particular after 6 months. The adjusted hazard ratio was 0.50 (0.21–1.19) in the total period, and 0.19 (0.04–0.85) in the period after 6 months.

The percentages of occurrence of one or more miscarriages were the same for both groups. Three BV qPCR negative tested persons experienced two or more miscarriages. One BV qPCR positive tested person experienced three miscarriages.

To study effect of microbiome community state types (CST) on time to live birth pregnancy rates, 16S rRNA gene microbiota analysis was performed on vaginal swabs obtained at IFA for 72 of these 82 participants. Of the 72 tested participants, 18, 3, 33, 17, and 1 participants were classified as CST I, II, III, IV, and V, respectively (Supplemental Table S1). A significant negative effect on time to live birth pregnancy rates was observed in CST group III dominated by *L. iners* (HR 0.45, 0.22–0.96) and CST group IV dominated by non-lactobacilli (HR 0.39, 0.16–0.98). Here, numbers were too low to perform accurate adjusting for BMI and age. A significant longer time to live birth pregnancy interval was observed for CST III and IV combined compared to CST I-II-V combined (Fig. 3b).

## Discussion

This is the first study to provide more insight into time to pregnancy interval in couples attending a fertility clinic, based on qPCR and microbiota testing. This study did not find an association between BV and time to live birth pregnancy in a general group of subfertile couples or in unexplained subfertility. In the subgroup of couples with an indication for IUI of IVF/ICSI, BV or an abnormal microbiota is possibly associated with a longer time to live birth pregnancy interval.

The effect of BV qPCR positivity on time to pregnancy was only observed after 6 months. This can be explained by the fact that it usually takes a few months to start IUI of IVF/ICSI treatment, and the couples who did not conceive spontaneously during this period have a poorer prognosis. Previous studies in IVF, focusing on the microbiome, have not conducted survival analyses before. The use of survival analysis is essential to gain better understanding of the implications of an abnormal microbiome in relation to pregnancy outcomes.

The follow-up period after start of pregnancy was long enough to report live birth rates, and there were no losses to follow-up among known pregnant participants, which are strengths of this study. Another strength of this study is that microbiome analysis is additionally used in a subgroup to enhance comparability of results with future studies.

This study could be influenced by information bias of the follow-up survey. Ten participants did not reply to our survey, so the follow-up time for these participants ended at their last visit or contact in the hospital, when they were not pregnant. Another limitation of this study is the small study size, especially of the subgroups. It was designed as part of a prospective cohort study about IUI and IVF/ICSI treatment (van den Tweel, 2023) [15], so no power analysis was conducted upfront and microbiome analysis was not available for every participant.

A study showed a high incidence of an endometrial Gardnerella biofilm in cases of non-viable pregnancy curettage [19]. Also a meta-analysis indicated more early pregnancy losses in BV-positive persons [9]. This was not observed in our study, but some indication of more miscarriages was observed in the subgroup with unexplained infertility (26% vs. 17%, *p* = 0.37). Experiencing a miscarriage could lead to delay in trying to conceive for a subsequent pregnancy. In our study, BMI was significantly larger for participants with BV. Obesity is associated with a deterioration in the vaginal microbiome, with reduced *Lactobacillus* dominance and increased diversity of bacteria [20]. A higher BMI has been associated with a longer time to pregnancy interval, so it could be confounding this study [21]. However, when adjusting for BMI, it did not change the hazard ratios.

There is a possibility that factors such as BV in unexplained subfertility could lead to better management of treating unexplained subfertility in the future. Even though this study had a limited number of persons with unexplained subfertility to provide a definite answer, we found a suggestive negative effect of BV in this group. More research in this specific group could lead to better understanding of why these couples do not timely conceive. Hopefully, in the future, these persons could be treated with more personalized medicine such as lifestyle interventions or for example vaginal microbiome transplantation.

As microbiota testing becomes more widely known, couples can ask for commercially available tests during their fertility treatment. This study could offer couples an answer, eliminating the need to test for BV at their initial fertility assessment. It could be a cost-effective indicator to check only the vaginal pH value at IFA if couples want to know about asymptomatic BV or are willing to try lifestyle interventions [22]. For persons starting with fertility treatments, BV qPCR testing could be considered to optimize treatment. It is shown in literature that the microbiome can change over time, but optimal timing of testing for BV is not yet known (at IFA or during fertility treatments). However, as there are currently no treatment options available for CST III and no evidence of improved pregnancy outcomes by treating CST IV, microbiota testing should at this moment be preserved for study purpose only.

### Supplementary Information

Below is the link to the electronic supplementary material.

Supplementary file1 (DOCX 2421 KB)

## Data Availability

The data underlying this article will be shared on reasonable request to the corresponding author.

## References

[1] Wang R, Danhof NA, Tjon-Kon-Fat RI, Eijkemans MJ, Bossuyt PM, Mochtar MH (2019). Interventions for unexplained infertility: a systematic review and network meta-analysis. Cochrane Database Syst Rev.

[2] Bourrion B, Panjo H, Bithorel P-L, de La Rochebrochard E, François M, Pelletier-Fleury N (2022). The economic burden of infertility treatment and distribution of expenditures overtime in France: a self-controlled pre-post study. BMC Health Serv Res.

[3] Boedt T, Vanhove A-C, Vercoe MA, Matthys C, Dancet E, Lie LF (2021). Preconception lifestyle advice for people with infertility. Cochrane Database Syst Rev.

[4] Hunault CC, Habbema JDF, Eijkemans MJC, Collins JA, Evers JLH, te Velde ER (2004). Two new prediction rules for spontaneous pregnancy leading to live birth among subfertile couples, based on the synthesis of three previous models. Hum Reprod.

[5] Spandorfer SD, Neuer A, Giraldo PC, Rosenwaks Z, Witkin SS (2001). Relationship of abnormal vaginal flora, proinflammatory cytokines and idiopathic infertility in women undergoing IVF. J Reprod Med.

[6] Babu G, Singaravelu BG, Srikumar R, Reddy SV, Kokan A (2017). Comparative study on the vaginal flora and incidence of asymptomatic vaginosis among healthy women and in women with infertility problems of reproductive age. J Clin Diagn Res.

[7] Borgdorff H, van der Veer C, van Houdt R, Alberts CJ, de Vries HJ, Bruisten SM (2017). The association between ethnicity and vaginal microbiota composition in Amsterdam, the Netherlands. Fredricks DN, editor PLoS One..

[8] van den Munckhof EHA, van Sitter RL, Boers KE, Lamont RF, Te Witt R, le Cessie S (2019). Comparison of Amsel criteria, nugent score, culture and two CE-IVD marked quantitative real-time PCRs with microbiota analysis for the diagnosis of bacterial vaginosis. European j clinic microbi & infect diseases: official publication of the European Society of Clinical Microbiology.

[9] Skafte-holm A, Humaidan P, Bernabeu A, Lledo B, Jensen JS, Haahr T (2021). The association between vaginal dysbiosis and reproductive outcomes in sub-fertile women undergoing ivf-treatment: a systematic PRISMA review and meta-analysis. Pathogens.

[10] Lokken EM, Manhart LE, Kinuthia J, Hughes JP, Jisuvei C, Mwinyikai K (2021). Association between bacterial vaginosis and fecundability in Kenyan women planning pregnancies: a prospective preconception cohort study. Hum Reprod.

[11] Hong X, Zhao J, Yin J, Zhao F, Wang W, Ding X (2022). The association between the pre-pregnancy vaginal microbiome and time-to-pregnancy: a Chinese pregnancy-planning cohort study. BMC Med.

[12] Bradshaw CS, Morton AN, Hocking J, Garland SM, Morris MB, Moss LM (2006). High recurrence rates of bacterial vaginosis over the course of 12 months after oral metronidazole therapy and factors associated with recurrence. J Infect Dis.

[13] Murphy K, Mitchell CM (2016). The interplay of host immunity, environment and the risk of bacterial vaginosis and associated reproductive health outcomes. J Infect Dis.

[14] Koedooder R, Singer M, Schoenmakers S, Savelkoul PHM, Morré SA, de Jonge JD (2019). The vaginal microbiome as a predictor for outcome of in vitro fertilization with or without intracytoplasmic sperm injection: a prospective study. Hum Reprod.

[15] van den Tweel MM, van den Munckhof EHA, van der Zanden M, Molijn AC, van Lith JMM, Le Cessie S, Boers KE (2024). Bacterial vaginosis in a subfertile population undergoing fertility treatments: a prospective cohort study. J Assist Reprod Genet.

[16] Nouioui I, Carro L, García-López M, Meier-Kolthoff JP, Woyke T, Kyrpides NC (2018). Genome-based taxonomic classification of the phylum actinobacteria. Front Microbiol.

[17] Ravel J, Gajer P, Abdo Z, Schneider GM, Koenig SSK, McCulle SL (2011). Vaginal microbiome of reproductive-age women. Proc Natl Acad Sci U S A.

[18] Walker AW, Martin JC, Scott P, Parkhill J, Flint HJ, Scott KP (2015). 16S rRNA gene-based profiling of the human infant gut microbiota is strongly influenced by sample processing and PCR primer choice. Microbiome.

[19] Swidsinski A, Verstraelen H, Loening-Baucke V, Swidsinski S, Mendling W, Halwani Z (2013). Presence of a polymicrobial endometrial biofilm in patients with bacterial vaginosis. PLoS ONE.

[20] Garg A, Ellis LB, Love RL, Grewal K, Bowden S, Bennett PR (2023). Vaginal microbiome in obesity and its impact on reproduction. Best Pract Res Clin Obstet Gynaecol.

[21] Loy SL, Cheung YB, Soh SE, Ng S, Tint MT, Aris IM, Bernard JY, Chong YS, Godfrey KM, Shek LP, Tan KH, Lee YS, Tan HH, Chern BSM, Lek N, Yap F, Chan SY, Chi C, Chan JKY (2018). Female adiposity and time-to-pregnancy: a multiethnic prospective cohort. Hum Reprod.

[22] van den Tweel MM, van der Struijs S, van den Munckhof EHA, Boers KE (2022). The relationship between vaginal pH and bacterial vaginosis as diagnosed using qPCR in an asymptomatic subfertile population. Arch Gynecol Obstet.

