# Supplementary material for: Testing on bacterial vaginosis in a subfertile population and time to pregnancy: a prospective cohort study
Source: Arch Gynecol Obstet. 2024 May 16;310(2):1245–53. doi: 10.1007/s00404-024-07542-x (PMC11258098; doi:10.1007/s00404-024-07542-x)
Supplement: Supplementary file 1 — Supplementary file1 (DOCX 2421 KB) [file 404_2024_7542_MOESM1_ESM.docx]

# Testing on bacterial vaginosis in a subfertile population and time to pregnancy: a prospective cohort study

**Drs. M.M. van den Tweel^ab^, dr. E.H.A. van den Munckhof^c^, dr. M. van der Zanden^b^, prof. S. Le Cessie^d,e^, prof. J.M.M. van Lith^a^, dr. K.E. Boers^b^**

^a^Department of Obstetrics and Gynecology, Leiden University Medical Center, 2300RC Leiden, The Netherlands; ^b^Department of Obstetrics and Gynecology, Haaglanden Medical Center, 2597AX The Hague, The Netherlands; ^c^ DDL Diagnostic Laboratory, 2288ER Rijswijk, The Netherlands ^d^Department of Biomedical Data Sciences, Leiden University Medical Center, 2300RC Leiden, The Netherlands, ^e^Department of Clinical Epidemiology, Leiden University Medical Center, 2300RC Leiden, the Netherlands

***Corresponding author:*** Dr. K.E. Boers, Haaglanden Medical Center, Bronovolaan 5, 2597 AX The Hague, The Netherlands. Phone number secretary K.E. Boers: + 31 88 979 4496. Email: kim.boers@haaglandenmc.nl.

# Attachment 1

Detailed description of microbiota analysis

A fragment of ~421bp of the V1-V2 region of the 16S rRNA gene was amplified using the primers described by Ravel, et al. (2011) and Walker, et al. (2015) with Illumina overhang adaptor sequences added. Each 50 µL PCR reaction contained 5 µL (10x) Expand High Fidelity Buffer with 15 mM MgCl2 (Roche), 2.6 U Expand High Fidelity Enzyme mix (Roche), 0.2 mM of each dNTP (Roche), various primer concentrations and 10 µL of extracted DNA. The PCR was run for 2 min at 94°C followed by 35 cycles of 94°C for 15 sec, 55 °C for 30 sec and 72 °C for 1 min and a final extension step at 72 °C for 7 min. The PCR products with a visible band of ~421bp on gel were subsequently purified and quantified using AMPure XP Beads (Agencourt Bioscience Corporation, Beverly, USA) and the Quant-iT PicoGreen dsDNA Assay Kit (Invitrogen, Paisley, UK), respectively. After library preparation using the Nextera XT kits (Illumina, San Diego, USA), sequencing was performed with the MiSeq desktop sequencer using the MiSeq Reagent Kits v2 500-cycles (Illumina). In each run, a negative control (PBS) and a positive control (Microbial Community Standard of ZymoBIOMICS) was included to monitor quality of the procedure. Samples should have a minimum number of 80,000 reads per sample and at least 75% of the reads should have an average quality score (Phred) ≥ Q30 to continue with data analysis. Sequencing data was processed following the QIIME pipeline(21) Open reference operational taxonomic units clustering of high-quality sequences (≥ 100bp in length with a quality score ≥ Q20) was conducted at a 97% similarity level against a pre-clustered version of the Augustus 2013 GreenGenes database. No low abundance filtering was used. Instead OTUs were checked for relevance per sample. Low abundance OTUs that were not relevant for any sample were included in the group “others”.

Determining the CST-classification

The highest percentage of type of Lactobacillus was chosen to determine to which CST the sample belonged. For example, if a sample contained 70% of *L. iners* and 20% of *L. jensenii*, the sample was classified to CST III (*L. iners*). Samples having less than 50% *Lactobacilli* were classified as CST IV.

| **Supplemental table 1** |  |  |  |  |  |  |
| --- | --- | --- | --- | --- | --- | --- |
| **Outcomes** | **CST I** | **CST II** | **CST III** | **CST IV** | **CST V** |  |
| **Persons starting IUI/IVF** | 18 | 3 | 33 | 17 | 1 |  |
| **Ongoing pregnancy,** n(%) | 14 (77%) | 2 (66%) | 16 (48%) | 7 (41%) | 1 |  |
| **Live birth** | 14 (77%) | 2 (66%) | 14 (42%) | 7 (41%) | 1 |  |
| **Premature birth** | 4 | 0 | 1 | 2 | 0 |  |
|  |  |  |  |  |  |  |
|  |  |  |  |  |  |  |
| **Supplemental table 2** | **Ongoing pregnancy** |  | **Live birth** |  | **Live birth** |  |
| **Coxregression** | **HR (CI 95%)** | **p-value** | **HR (CI 95%)2** | **p-value2** | **aHR (CI 95%)**** | **p-value3** |
| **CST I** | 1 |  | 1 |  |  |  |
| **CST II** | 0.69 (0.16-3.03) | 0.62 | 0.69 (0.16-3.06) | 0.63 | 0.50 (0.11-2.27) | 0.37 |
| **CST III** | 0.52 (0.25 -1.07) | 0.08 | 0.45 (0.22 -0.96) | **0.04*** | 0.45 (0.21-0.96) | **0.04*** |
| **CST IV** | 0.40 (0.16-1.00) | **0.05*** | 0.39 (0.16-0.98) | **0.05*** | 0.34 (0.13-0.90) | **0.03*** |
| **CST V** | 1.63 (0.21-12.60) | 0.64 | 1.71 (0.22-13.22) | 0.61 | 3.19 (0.38-26.99) | 0.29 |
|  |  |  |  |  |  |  |
|  |  |  |  |  |  |  |
| **Supplemental table 3** |  |  |  |  |  |  |
| **Time to live birth pregnancy** | **BV neg** | **BV pos** | **HR (CI 95%)** | **p-value** | **aHR (CI 95%)**** | **p-value2** |
| Caucasian descent | 78 | 22 | 1.22 (0.64-2.31) | 0.55 | 1.24 (0.63-2.45) | 0.54 |
| non-Caucasian descent | 39 | 21 | 0.98 (0.42-2.31) | 0.97 | 0.90 (0.39-2.11) | 0.81 |
|  |  |  |  |  |  |  |
| * p-value < 0.05 considered significant | |  |  |  |  |  |
| **adjusted for BMI and age |  |  |  |  |  |  |

Supplemental table 1: Description of pregnancy results per community state type (CST)

Supplemental table 2: Pregnancy results by CST, CST I (*L.crispatus*) used as reference CST

Supplemental table 3: Pregnancy results by qPCR based on ethnicity


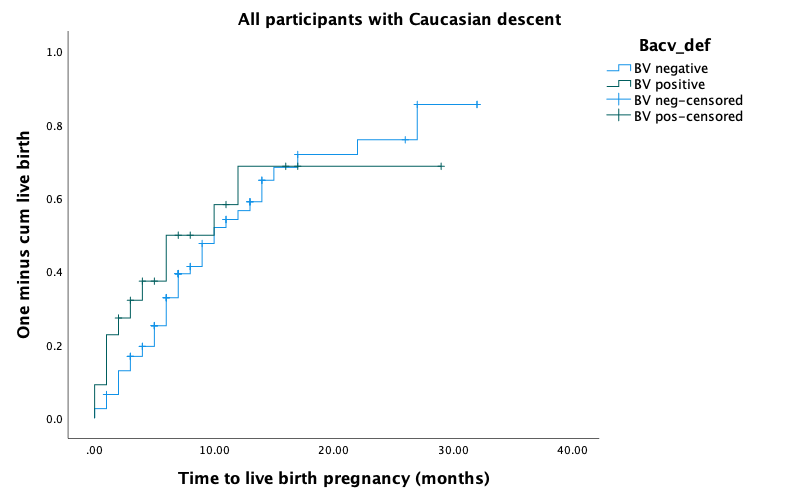

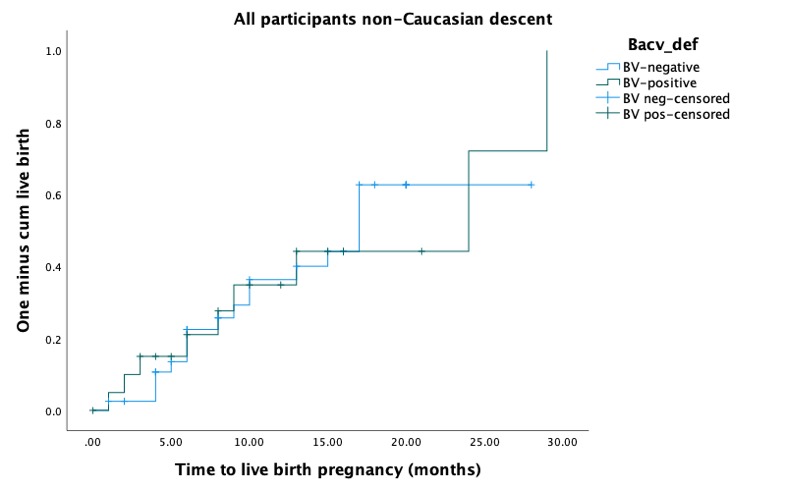
**Fig. 4**: Kaplan Meier curves for time to live birth pregnancy by BV qPCR for all participants at IFA with (a) Caucasian descent and (b) non-Caucasian descent
